# Determinants of maternal overweight in a low-resourced setting in Ghana: a cross-sectional survey among pregnant women

**DOI:** 10.1186/s13104-025-07410-0

**Published:** 2025-10-09

**Authors:** Joseph Lasong, Yula Salifu, Torjim Salifu

**Affiliations:** Department of Population and Reproductive Health, School of Public Health, University for Development Studies, Tamale, Ghana

**Keywords:** Knowledge, Determinants, Overweight, Maternal, Rural, Ghana

## Abstract

**Objectives:**

Maternal overweight is an emerging public health concern in low-resource settings, where limited healthcare access and low awareness levels may hinder effective prevention. This study assessed the determinants of maternal overweight among pregnant women in the Gushegu District of Northern Ghana.

**Methods:**

A community-based cross-sectional survey was conducted among 377 pregnant women aged 18 years and above. Data were collected through structured interviews and from antenatal records. Key variables examined included socio-demographic factors, pregnancy-related characteristics, and awareness of maternal overweight. Logistic regression analysis was used to identify independent predictors.

**Results:**

The prevalence of maternal overweight was 37.1%. Women in their second trimester had significantly higher odds of being overweight compared to those in the third trimester (AOR = 2.99, 95% CI: 1.18–7.60; *p* = 0.021). Employment was also positively associated with overweight (unemployed: AOR = 0.18, 95% CI: 0.09–0.37; *p* < 0.001), while lack of prior awareness about maternal overweight was linked to reduced odds (AOR = 0.38, 95% CI: 0.17–0.83; *p* = 0.015).

**Conclusions:**

Maternal overweight was common in this rural setting with trimester of pregnancy, employment and awareness on overweight as predictors of maternal overweight. Strengthening antenatal education and awareness, particularly during early pregnancy, may help mitigate overweight-related risks among pregnant women.

## Introduction

Overweight is a growing global health concern in both high-income and emerging economies. Its prevalence has increased significantly over the past decades, posing serious risks to maternal and child health [1]. Globally, over 1.8 billion adults are classified as overweight, according to the World Health Organization [2]. This upward trend is mirrored in maternal populations, with a growing number of pregnant women presenting for obstetric care while overweight or obese [3]. In countries such as Australia and the United States, the proportion of overweight pregnant women has reached 50% and 36%, respectively [4].

In Ghana, the prevalence of maternal overweight and obesity varies widely, ranging from 7.5 to 50% depending on region and population subgroup [5–9]. A 20-year analysis of maternal health data from Germany also revealed a significant increase in maternal body mass index, with the proportion of women classified as obese rising from 9.4 to 19.2%, corresponding with an increase in average periconceptional weight from 67.6 kg to 72.0 kg [10]. In other parts of Africa, Cheng et al. reported maternal obesity prevalence ranging from 6.5 to 50.7% [11]. Similar increasing trends have been documented globally: a cohort study in Accra, Ghana, found that 48.2% of women were overweight or obese (31.3% overweight; 16.9% obese) [12], while figures of 46% and 16–33% were reported among pregnant women in Wales and across 48 U.S. states, respectively [13, 14].

Despite these alarming statistics, preventive and control measures remain underutilized, particularly among at-risk populations [3]. Contributing risk factors include dietary excess, sedentary lifestyles, and low levels of maternal health literacy [3, 15, 16]. Poor nutrition knowledge [17] and insufficient physical activity [18] also compound the risk of maternal overweight. Importantly, overweight during pregnancy is an independent risk factor for several complications, including gestational diabetes, hypertensive disorders, and adverse neonatal outcomes [19]. It is also associated with longer time to conception and higher likelihood of long-term cardiometabolic conditions among affected women [20, 21]. Interventions targeting overweight in women of reproductive age can substantially reduce pregnancy-related complications and improve maternal outcomes [22].

Understanding the determinants of maternal overweight is critical for guiding antenatal counseling, nutrition interventions, and public health policies. However, there remains a paucity of research on this issue in Ghana, especially in rural and underserved areas. Gushegu District, located in northern Ghana, is characterized by limited healthcare infrastructure, increased poverty and food insecurity, and low levels of education [23, 24]. The extent and drivers of maternal overweight in such settings remain poorly documented. Therefore, this study aimed to assess the determinants of maternal overweight among pregnant women in the Gushegu District of Northern Ghana.

## Methods

### Study design and setting

A community-based cross-sectional study was conducted to examine the determinants of maternal overweight in the Gushegu District of Northern Ghana. This design was selected for its ability to evaluate associations between exposures and outcomes at a specific point in time [25].

The study took place in Gushegu District, located in the northeastern part of Ghana’s Northern Region. The district comprises five sub-districts and has Gushegu as its capital. It is classified as a low-resourced area due to limited healthcare infrastructure, high poverty rates (with over 60% of the population living below the national poverty line), low antenatal care coverage, and a predominantly rural population [26]. According to the 2010 Ghana Population and Housing Census, the district had a total population of 111,259, including 54,186 males and 57,073 females [26].

### Study population and eligibility criteria

The study targeted pregnant women residing in Gushegu District between June and July 2021. Inclusion criteria were being aged 18 years or older, having an antenatal record book, being present during the study period, having a history of live pregnancy within the last 5 years and providing written informed consent. Women were excluded if they had multiple pregnancies, were underweight or obese, were unable to communicate independently, had gestational diabetes, physical impairments, or self-reported or clinically diagnosed mental illness. Women with underweight and obesity were excluded to focus specifically on overweight as an outcome and to minimize confounding due to distinct risk profiles. Exclusion of women with medical complications or multiple pregnancies was intended to reduce variability in pregnancy-related weight gain.

### Sample size and recruitment strategy

The sample size was calculated using Cochrane’s formula for cross-sectional studies:

N ≥ Z²p(1 − p)/d², where Z is the z-score at 95% confidence level (1.96), p is the estimated prevalence of overweight (29%) [26], and d is the margin of error (5%). This yielded a minimum required sample size of 316. After accounting for a 10% non-response rate, a final sample size of 348 was achieved. Of 414 women approached, 377 participated, resulting in a response rate of 91.1%.

A multistage sampling approach was employed. First, one community was randomly selected from each of the five sub-districts. Within each selected community, the sample size was proportionally allocated based on estimated reproductive-aged population figures. Households were systematically selected using a sampling interval of four, and one eligible pregnant woman per household was selected through simple random sampling if more than one was present. If a selected household did not contain an eligible participant, the next household was approached. This was done until all eligible respondents were sampled. Interviews were conducted in private settings to ensure confidentiality and comfort.

### Measurement of variables

The outcome variable was maternal overweight, defined by body mass index (BMI). BMI was calculated using height and weight recorded in antenatal record books during the first clinic visit (booking weight and height). BMI categories followed WHO classification: underweight (< 18.5 kg/m²), normal weight (18.5–24.9 kg/m²), overweight (25.0–29.9 kg/m²), and obese (≥ 30.0 kg/m²) [27]. For analysis, BMI was dichotomized as normal weight versus overweight. Gestational age adjustments were not applied.

Independent variables included:

Socio-demographic variables: age, education, employment, occupation, family history of overweight.

Obstetric factors: parity, trimester, place of delivery, mode of delivery, and pre-existing medical conditions.

Knowledge variables: prior awareness of maternal overweight, perceived causes, and beliefs about weight gain during pregnancy.

The questionnaire was adapted from literature [1, 5–9, 15–17], pre-tested, and reviewed by maternal health experts. It was administered in English or a local language (e.g., Dagbani), depending on respondent preference. The study collected quantitative data only. While no composite knowledge score was computed, individual items related to knowledge were modelled as separate predictors in the regression analysis.

### Data quality control

Data quality was ensured through pre-testing of instruments in a non-study community nearby, which shares similar characteristics with the study location, daily supervision of fieldwork, double entry of data and consistency checks and training of research assistants in research ethics and survey techniques. Research assistants were medical professionals of any cadre with an MSc, MA, MPhil, MPH or any medical, public health and allied health master’s degree with a minimum of 3 years clinical and/or research experience. The research assistants were trained for 4 days for the data collection process. All data were put under lock and key and secured with a password on a computer with only the principal investigator having access to it.

Each interview lasted approximately 20 to 45 min. The study adhered to the Strengthening the Reporting of Observational Studies in Epidemiology (STROBE) guidelines. Ethical approval was obtained from the University for Development Studies Ethics Review Committee. All participants provided written informed consent. The study followed the ethical principles outlined in the Declaration of Helsinki (2013 revision), ensuring voluntary participation, confidentiality, and anonymity.

### Data analysis

Data were entered and analysed using IBM SPSS Statistics version 25. Descriptive statistics (means, standard deviations, and percentages) summarized participant characteristics. Bivariable analyses were conducted using Chi-square tests to identify associations between the outcome and predictor variables. Variables with a p-value < 0.1 in bivariable analysis were considered for inclusion in the multivariable model. Multicollinearity was assessed using the variance inflation factor (VIF), with a threshold of > 5 used to identify and exclude collinear variables. Model fit was assessed using the Hosmer–Lemeshow goodness-of-fit test (*p* > 0.05 indicating good fit). A multivariable logistic regression analysis was conducted using a backward stepwise elimination method to identify independent predictors of maternal overweight. Variables retained in the final model included trimester, education, employment, and awareness of overweight. A 5% level of significance was used in the final analysis.

## Results

### Socio-demographic characteristics of respondents

Table 1 presents the association between respondents’ socio-demographic characteristics and overweight status. Among the 377 participants, 37.1% were classified as overweight. The prevalence of overweight varied by trimester, with the highest proportion observed in the second trimester (65.2%), followed by the first (42.9%) and third (35.2%) trimesters.

Most respondents were aged 25 years or older (75.1%) and had two or fewer children (79.0%). Home delivery was reported by 11.1% of women, of whom 61.8% had spontaneous vaginal delivery. At the time of data collection, most participants (92.0%) were in their third trimester, and 97.9% had no reported pre-existing medical or gynaecological conditions. In terms of education and employment, 55.2% had some level of formal education, and 27.1% were unemployed. Regarding family history, 9.3% of respondents indicated that a female family member had experienced overweight during pregnancy, 2.1% reported general family overweight among women, and 28.0% observed excessive postpartum weight gain among female relatives.

Significant associations with BMI were found for pregnancy trimester (*p* = 0.011), educational status (*p* = 0.002), employment status (*p* < 0.001), and family history of overweight (*p* < 0.001). Although a slightly higher proportion of younger women (< 25 years) were overweight (37.2%) compared to their older counterparts (≥ 25 years), this difference was not statistically significant.

**Table 1:** Socio-demographic characteristics and association with overweight

Variable	Frequency (%)	BMI *N* (%)		*p*-value
		Normal weight	Overweight	
**Maternal Age (years)**	π (SD); 26.43 (3.41)			
< 25	94 (24.9)	59 (62.8)	35 (37.2)	1.000
≥ 25	283 (75.1)	178 (62.9)	105 (37.1)	
**Number of Children**	π (SD); 2.07 (0.69)			
≤ 2	298 (79.0)	189 (63.4)	109 (36.6)	0.195
> 2	79 (21.0)	48 (60.8)	31 (39.2)	
**Place of Delivery**				
Home	42 (11.1)	29 (69.0)	13 (31.0)	0.203
Hospital	335 (88.9)	208 (62.1)	127 (37.9)	
**Delivery Method**				
Caesarean Section	144 (38.2)	86 (59.7)	58 (40.3)	0.426
Spontaneous Vaginal Delivery	233 (61.8)	151 (64.8)	82 (35.2)	
**Trimester of Pregnancy**				
First	7 (1.9)	4 (57.1)	3 (42.9)	0.011
Second	23 (6.1)	8 (34.8)	15 (65.2)	
Third	347 (92.0)	225 (64.8)	122 (35.2)	
**Pre- Existing Medical/ Gynecological Condition**				
No	369 (97.9)	231 (62.6)	138 (37.4)	0.115
Yes	8 (2.1)	6 (75.0)	2 (25.0)	
**Educational Status**				
No Formal Education	169 (44.8)	121 (71.6)	48 (28.4)	0.002
Formal Education	208 (55.2)	116 (55.8)	92 (44.2)	
**Employment Status**				
Unemployed	102 (27.1)	87 (85.3)	15 (14.7)	< 0.001
Employed	275 (72.9)	150 (54.5)	125 **(**45.5)	
**Family member experience with overweight during pregnancy**				
No	342 (90.7)	219 (64.0)	123 (36.0)	0.146
Yes	35 (9.3)	18 (51.4)	17 (48.6)	
**Family overweight among women**				
No	369 (97.9)	237 (64.2)	132 (35.8)	< 0.001
Yes	8 (2.1)	0 (0.0)	8 (100.0)	
**Postpartum overweight among family members**				
No	268 (71.1)	171 (63.8)	97 (36.2)	0.559
Yes	109 (28.9)	66 (60.6)	43 (39.4)	
**Body mass index**				
Normal weight	237 (62.9)			
Overweight	140 (37.1)			

π (SD) = Mean (Standard Deviation), N = Frequency, %=Percentage, BMI = Body Mass Index

### Knowledge of respondents on overweight

Table 2 presents the association between respondents’ knowledge and overweight. Overall, 87.8% of the participants reported having prior knowledge of overweight in pregnancy. Of these, only 13.5% had received information from informal sources such as the media, peers, or friends. A large majority (90.7%) believed that overweight could be inherited, while 50.7% identified an uncontrolled diet as the primary cause. Additionally, 16.0% of respondents felt that gaining weight during pregnancy was necessary. Bivariable analysis revealed that having prior knowledge of overweight in pregnancy was significantly associated with overweight status (*p* = 0.009).

**Table 2:** Overweight knowledge and association with overweight

Variable	Frequency (%)	BMI *N* (%)		*p*-value
		Normal weight	Overweight	
**Ever heard of overweight in pregnancy**				
No	46 (12.2)	37 (80.4)	9 (19.6)	0.009
Yes	331 (87.8)	200 (60.4)	131 (39.6)	
**Mode of awareness about maternal overweight**				
Media/peers/friends	51 (13.5)	27 (52.9)	24 (47.1)	0.276
Health professional/family member	280 (74.3)	173 (61.8)	107 (38.2)	
**Maternal Overweight Can Be Inherited**				
No	342 (90.7)	219 (64.0)	123 (36.0)	0.146
Yes	35 (9.3)	18 (51.4)	17 (48.6)	
**Causes of Overweight During Pregnancy**				
Uncontrolled diet	191 (50.7)	118 (61.8)	73 (38.2)	0.753
Lack of exercise	133 (35.3)	87 (65.4)	46 (34.6)	
Too much resting	53 (14.1)	32 (60.4)	21 (39.6)	
**Weight gain during pregnancy is Necessary**				
No	316 (83.8)	196 (62.0)	120 (38.0)	0.173
Yes	61 (16.2)	41 (67.2)	20 (32.8)	

N = Frequency, %= Percentage, BMI = Body Mass Index

### Determinants of maternal overweight

Table 3 presents the factors associated with maternal overweight. Compared to women in their third trimester, those in the second trimester had significantly higher odds of being overweight (AOR = 2.99, 95% CI: 1.18–7.60; *p* = 0.021). Women in the first trimester, however, did not differ significantly from those in the third trimester (AOR = 1.35, 95% CI: 0.28–6.65; *p* = 0.710). With respect to employment status, unemployed women had significantly lower odds of being overweight compared to employed women (AOR = 0.18, 95% CI: 0.09–0.37; *p* < 0.001). In terms of awareness, women who had never heard of overweight had significantly lower odds of being overweight compared to those who had prior awareness (reference group) (AOR = 0.38, 95% CI: 0.17–0.83; *p* = 0.015).

**Table 3:** Logistic regression of the determinants of overweight

Variable	Categorization	AOR	CI (95%)	*P*-Value
**Trimester of Pregnancy**	First Trimester	1.353	0.275–6.647	0.710
	Second Trimester	2.994	1.180–7.595	0.021
	Third Trimester	Reference		
**Employment Status**	Unemployed	0.178	0.086–0.368	<0.001
	Employed	Reference		
**Ever heard of weight gain and obesity in pregnancy or after delivery**	No	0.376	0.171–0.826	0.015
	Yes	Reference		

AOR = Adjusted Odds Ratio, CI (95%) = 95% Confidence Interval. Family overweight, educational status, awareness of overweight, trimester of pregnancy and employment status were entered in the logistic regression model

## Discussion

Maternal overweight is a growing global health concern with significant implications for maternal and neonatal outcomes [28]. Once considered a problem limited to high-income countries, the burden of maternal overweight has sharply increased in low- and middle-income countries (LMICs) [29], possibly due to rapid urbanization, nutrition transition, and limited maternal health literacy [30, 31]. The present study found a prevalence of 37.1% for maternal overweight in a rural district (Gushegu district) in Northern Ghana, aligning with trends observed across similar contexts. This prevalence is comparable to findings from United States (36%) [4], yet lower than those reported in urban Ghana (48.2%) [12] and Australia (50%) [4].

However, other studies in Ghana report even wider ranges, from 7.5 to 50% depending on geographic, socioeconomic, and health service access differences [5–9]. The lower prevalence in some rural areas may reflect less access to calorie-dense foods and more physically demanding lifestyles. In contrast, urban populations are often exposed to sedentary occupations and ultra-processed food consumption, which may contribute to higher rates of overweight. A 20-year analysis in Germany, for example, demonstrated a rise in average preconception BMI from 67.6 kg to 72.0 kg, with obesity rates nearly doubling [10]. These trends underscore the global nature of the problem, while emphasizing context-specific determinants in different populations. The findings further suggest the urgent need for national and district-level nutritional surveillance systems that can detect and track maternal overweight trends, particularly in rural areas often excluded from most urban-biased health policies. These insights are critical for shaping antenatal interventions in low-resource settings.

In the current study, trimester of pregnancy emerged as a significant determinant of maternal overweight. Women in their second trimester had almost three times the odds of being overweight compared to those in the third trimester. This is consistent with literature indicating that the second trimester is a period of rapid physiological changes, including expansion of plasma volume, extracellular fluid, and amniotic fluid, along with accelerated foetal and placental growth [1, 2, 32]. These factors contribute to an increase in maternal weight that may not reflect actual fat accumulation, particularly when BMI is calculated based on booking weight rather than pre-pregnancy values. As such, BMI measurements during pregnancy, especially in the second and third trimesters, should be interpreted with caution due to potential overestimation related to fluid retention [32]. Nevertheless, pre-pregnancy BMI or early gestational weight data should be routinely collected to improve risk stratification and clinical decision-making. Also, training health workers on gestational physiology and appropriate weight monitoring should be incorporated into routine antenatal service protocols.

Employment status was also significantly associated with overweight. Employed women were more likely to be overweight than their unemployed counterparts, a finding supported by studies in Ethiopia and Ghana [6, 8]. Employed women, particularly in sedentary or market-based occupations, may have reduced opportunities for physical activity and greater access to energy-dense, processed foods. These behaviours are typical of nutrition transitions in LMICs, where rising socioeconomic status often leads to adoption of Westernized diets and reduced energy expenditure [7]. Conversely, unemployed women may engage in more physically active domestic tasks and have limited access to obesogenic foods, possibly explaining the inverse association. Thus, public health interventions should target employed women with tailored messages on meal planning, physical activity, and healthy weight management during pregnancy. Also, workplace-based antenatal education and nutritional counselling programs should be developed, especially in informal and market-based employment sectors.

Interestingly, this study found that women who had never heard of maternal overweight were significantly less likely to be overweight. While initially counterintuitive, this finding may reflect complex interactions between awareness, health behaviour, and structural inequities in health education. In low-literacy and rural populations, limited exposure to antenatal health messages may lead to misperceptions about pregnancy weight gain. Studies in Ghana and South Asia have noted poor awareness and widespread misconceptions about healthy weight gain in pregnancy, particularly among women with low education levels and limited antenatal care attendance [9, 15, 16]. In such contexts, weight gain may be viewed as a sign of a healthy pregnancy, and the concept of “overweight” may not be well understood. This highlights the need for targeted, culturally sensitive health education to improve knowledge and reshape perceptions. Moreso, health education strategies must address rural-specific misconceptions about pregnancy weight and body image through culturally adapted content.

Contrary to some studies, this research did not find a significant association between maternal age and overweight, although a slightly higher proportion of women under 25 were overweight. While older maternal age is often associated with higher BMI due to reduced metabolism and increased fat deposition [33], findings across sub-Saharan Africa remain inconsistent. Studies in Cape Coast [34] and Southern Ghana [35] have shown higher overweight prevalence among older women, while others have reported similar trends among younger women. These inconsistencies may reflect differences in reproductive history, activity levels, and cultural norms across regions. Age-specific antenatal counseling should be included in national antenatal care guidelines, as both young and older pregnant women may benefit from tailored guidance on healthy weight gain. Further, health workers should conduct BMI risk assessments across all age groups during antenatal visits and adapt counselling accordingly.

### Strengths and limitations

This study contributes to the growing body of knowledge on gestational overweight and its associated risk factors, particularly in rural, low-resource settings. Also, focusing on pregnant women in Northern Ghana, a population often underrepresented in maternal health research, provides important insights into the burden of maternal overweight and supports the development of community-level interventions. The findings may also inform policy discussions on maternal nutrition and health promotion, aligning with global efforts to achieve Sustainable Development Goal 3: Good Health and Well-being. Notably, the use of a community-based design with a high response rate strengthens the internal validity of the study. The focus on a rural and underserved population enhances its relevance for targeted maternal health strategies in similar contexts.

However, the study has several limitations. First, BMI measurements during pregnancy may be affected by physiological changes, particularly fluid retention in the second and third trimesters, which may not accurately reflect pre-pregnancy nutritional status. Second, the cross-sectional design limits the ability to draw causal inferences. Third, reliance on self-reported knowledge introduces the potential for recall and response bias. Additionally, the relatively modest sample size may limit the statistical power and generalizability of findings beyond rural settings, especially to urban populations with different socioeconomic and healthcare dynamics. Therefore, results should be interpreted with caution, and further research using longitudinal or mixed-methods approaches is recommended.

### Conclusions and recommendations

Maternal overweight remains a significant public health issue in low-resource, rural areas of Ghana. The study identified key factors associated with overweight during pregnancy, including trimester, employment status, and awareness of maternal weight-related risks. To reduce overweight-related complications, health education efforts must be strengthened during antenatal care. Pregnant women should be supported to set appropriate gestational weight gain goals, with personalized counselling on nutrition and physical activity. Regular monitoring of BMI during pregnancy and tailored guidance on healthy lifestyle choices should be incorporated into routine antenatal services.

Given the global burden of obstetric complications linked to maternal overweight, interventions should focus on bridging knowledge gaps through community engagement, targeted health sensitization campaigns, and nutrition education at the household and facility levels. Antenatal care protocols should integrate weight management counselling as a standard component of maternal care. While this study highlights key associations, it does not establish causal relationships. Future research should adopt robust epidemiological designs, including prospective cohort studies and qualitative methods, to explore the personal, cultural, and community perceptions of gestational overweight. Such insights will be crucial in designing culturally appropriate and sustainable interventions to reduce the burden of maternal overweight in similar low-resource settings.

## Data Availability

The datasets used and/or analyzed during the current study are contained in the manuscript.

## Abbreviations

AOR: Adjusted Odds Ratio
BMI: Body Mass Index
CI (95%): 95% Confidence Interval
GDHS: Ghana Demographic and Health Survey
LMICs: Low-Middle-Income Countries
OW: Overweight
SPSS: Statistical Package for Social Sciences
WHO: World Health Organization
